# Contrast-Enhanced Ultrasound with VEGFR2-Targeted Microbubbles for Monitoring Regorafenib Therapy Effects in Experimental Colorectal Adenocarcinomas in Rats with DCE-MRI and Immunohistochemical Validation

**DOI:** 10.1371/journal.pone.0169323

**Published:** 2017-01-06

**Authors:** Ralf Stefan Eschbach, Dirk-Andre Clevert, Heidrun Hirner-Eppeneder, Michael Ingrisch, Matthias Moser, Jessica Schuster, Dina Tadros, Moritz Schneider, Philipp Maximilian Kazmierczak, Maximilian Reiser, Clemens C. Cyran

**Affiliations:** 1 Laboratory for Experimental Radiology, Institute for Clinical Radiology, Ludwig-Maximilians-University Hospital, Munich, Germany; 2 Josef Lissner Laboratory for Biomedical Imaging, Institute for Clinical Radiology, Ludwig-Maximilians-University Hospital, Munich, Germany; Universidade de Sao Paulo, BRAZIL

## Abstract

**Objectives:**

To investigate contrast-enhanced ultrasound (CEUS) with VEGFR2-targeted microbubbles for monitoring therapy effects of regorafenib on experimental colon carcinomas in rats with correlation to dynamic contrast-enhanced MRI (DCE-MRI) and immunohistochemistry.

**Materials and Methods:**

Human colorectal adenocarcinoma xenografts (HT-29) were implanted subcutaneously in n = 21 (n = 11 therapy group; n = 10 control group) female athymic nude rats (*Hsd*: *RH-Foxn1*^*rnu*^). Animals were imaged at baseline and after a one-week daily treatment with regorafenib or a placebo (10 mg/kg bodyweight), using CEUS with VEGFR2-targeted microbubbles and DCE-MRI. In CEUS tumor perfusion was assessed during an early vascular phase (wash-in area under the curve = WiAUC) and VEGFR2-specific binding during a late molecular phase (signal intensity after 8 (SI_8min_) and 10 minutes (SI_10min_)), using a conventional 15L8 linear transducer (transmit frequency 7 MHz, dynamic range 80 dB, depth 25 mm). In DCE-MRI functional parameters plasma flow (PF) and plasma volume (PV) were quantified. For validation purposes, CEUS parameters were correlated with DCE-MRI parameters and immunohistochemical VEGFR2, CD31, Ki-67 and TUNEL stainings.

**Results:**

CEUS perfusion parameter WiAUC decreased significantly (116,989 ± 77,048 a.u. to 30,076 ± 27,095a.u.; p = 0.005) under therapy with no significant changes (133,932 ± 65,960 a.u. to 84,316 ± 74,144 a.u.; p = 0.093) in the control group. In the therapy group, the amount of bound microbubbles in the late phase was significantly lower in the therapy than in the control group on day 7 (SI_8min_: 283 ± 191 vs. 802 ± 460 a.u.; p = 0.006); SI_10min_: 226 ± 149 vs. 645 ± 461 a.u.; p = 0.009). PF and PV decreased significantly (PF: 147 ± 58 mL/100 mL/min to 71 ± 15 mL/100 mL/min; p = 0.003; PV: 13 ± 3% to 9 ± 4%; p = 0.040) in the therapy group. Immunohistochemistry revealed significantly fewer VEGFR2 (7.2 ± 1.8 vs. 17.8 ± 4.6; p < 0.001), CD31 (8.1 ± 3.0 vs. 20.8 ± 5.7; p < 0.001) and Ki-67 (318.7 ± 94.0 vs. 468.0 ± 133.8; p = 0.004) and significantly more TUNEL (672.7 ± 194.0 vs. 357.6 ± 192.0; p = 0.003) positive cells in the therapy group. CEUS parameters showed significant (p < 0.05) correlations to DCE-MRI parameters and immunohistochemistry.

**Conclusions:**

CEUS with VEGFR2-targeted microbubbles allowed for monitoring regorafenib functional and molecular therapy effects on experimental colorectal adenocarcinomas with a significant decline of CEUS and DCE-MRI perfusion parameters as well as a significant reduction of specifically bound microbubbles under therapy, consistent with a reduced expression of VEGFR2.

## Introduction

Colorectal cancer (CRC) is one of the most prevalent cancers worldwide, as well as one of the most deadly. More than 1,200,000 people are diagnosed annually with CRC, and more than 600,000 die from CRC per year [1]. The World Health Organization (WHO) estimates an increase of 77% in the number of newly diagnosed cases of CRC and an increase of 80% in deaths from CRC by 2030 [2]. Angiogenesis has been demonstrated to play a pivotal role in CRC and overexpression of VEGF and high vascular density in primary CRCs are associated with an increased risk of tumor recurrence and the formation of metastases [1]. Regorafenib is an oral multi tyrosine kinase inhibitor that showed anti-angiogenic and anti-proliferative effects *in vivo* in several experimental tumor models, like breast cancer, renal cell carcinoma and glioblastoma [3]. Regorafenib has demonstrated a significant improvement in overall survival in a phase III study in patients with metastatic CRC who failed previous therapies [1]. It has subsequently become the first approved treatment for this indication by the US Food and Drug Administration (FDA) and European Medical Agency (EMA) [1,4,5].

While anti-angiogenic, not primarily cytotoxic tumor therapeutics such as regorafenib have been shown to exhibit significant effects on tumor angiogenesis and metabolism, only subtle effects were observed on tumor size and morphology, particularly in the first phase following therapy initiation [3,6]. In this context, approved methods of monitoring primarily cytotoxic cancer therapies, such as the morphology-based Response Evaluation Criteria in Solid Tumors (RECIST), are not adequately sensitive for a reliable monitoring of the early therapeutic effects of molecular anti-angiogenic therapeutics [3,6–8]. Alternatively, functional and molecular imaging biomarkers assessed by multiparametric imaging methods such as contrast-enhanced ultrasound (CEUS) may provide a sensitive tool for an early and reliable therapy monitoring of molecular, anti-angiogenic drugs. Multiparametric CEUS with targeted microbubbles (MB) can characterize morphological, functional and molecular parameters of tumor pathophysiology from its earliest stages [9–16]. Preclinical and clinical studies demonstrated the feasibility of functional and molecular CEUS imaging with VEGFR2 (vascular endothelial growth factor receptor 2) targeted MB in monitoring therapy response to anti-angiogenic treatment in different tumor entities [11,17–21], as VEGF is one of the most potent growth factors of endothelial cells and a main regulator of angiogenesis [22–24]. Following intravenous injection, in a first early phase tumor perfusion can be quantitatively evaluated by the assessment of various parameters of tissue microcirculation. In a second, late phase, targeted MB bound to tumor vascular endothelial cells, overexpressing VEGFR2 as molecular markers of angiogenesis, allow for the non-invasive visualization of VEGF receptor 2 expression *in vivo* [13,21]. Thereby CEUS with BR55 may be able to provide combined functional and molecular imaging biomarkers for the non-invasive assessment of tumor angiogenesis and therapy response of the investigated colon cancer xenografts under regorafenib therapy.

The hypothesis of this study is that CEUS with VEGFR2-targeted microbubbles allows for functional and molecular in *vivo* monitoring of regorafenib therapy effects in colorectal adenocarcinoma xenografts in rats. The purpose of this project was to investigate whether the acquired functional CEUS parameters of tumor microcirculation and the molecular CEUS parameters of VEGFR2-specific binding can be applied as non-invasive imaging biomarkers of therapy response and correlate it with DCE-MRI parameters of tumor microcirculation, to acquire a composite functional and molecular biomarker panel with complementary information of tumor angiogenesis, validated by immunohistochemistry as standard of reference.

## Materials and Methods

### Animal model and experimental protocol

**Fig 1:**
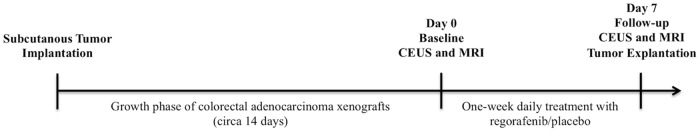
Timeline of the experimental set-up from tumor cell implantation to tumor explantation with immunohistochemical analysis.

This study was conducted in accordance with the guidelines for the care and use of laboratory animals of the German Federal Ministry of Food and Agriculture. The protocol was approved by the Committee for Animal Research of the Government of Upper Bavaria (Gz.55.2-1-54-2532-178-13). Human colorectal adenocarcinoma cells HT-29 (ATCC HTB-38, Manassas, VA) were resuspended in a total volume of 0.5 mL as a 1:1 mixture of phosphate buffered saline (PBS pH 7.4; GIBCO Life Technologies, Darmstadt, Germany) and Matrigel^®^ (BD Biosciences, San Jose, CA). 3 x 10^6^ cells per animal were injected subcutaneously into the left abdominal flank of a total of n = 21 (7–8 weeks old) female athymic nude rats (*Hsd*: *RH-Foxn1*^*rnu*^, Harlan Laboratories Inc., Indianapolis, IN). Tumor growth was assessed by daily caliper measurements in two dimensions. When subcutaneous tumor xenografts reached adequate size, animals were randomly assigned to either the therapy (n = 11) or to the control group (n = 10). For imaging, rats were anesthetized by continuous inhalation of isoflurane (2.5% in pure oxygen, 2 L/min) and placed on a heating pad to maintain physiological body temperature for the whole examinations. A multimodality imaging protocol with CEUS and subsequent DCE-MRI examination was performed on day 0 (baseline) and on day 7 (follow-up). Animals were treated daily with the multityrosine kinase inhibitor regorafenib (therapy group; 10 mg/kg body weight formulated in a solution 42.5/42.5/15 of polypropylen glycol/PEG400/pluronic F68 + 20% aqua, Bayer HealthCare, Leverkusen, Germany) or placebo (control group; volume equivalent applications of regorafenib solvent cremophor/ethanol) from day 1 to day 6 via gastric gavage using a dedicated 16-gauge curved buttoned cannula. After follow-up measurements on day 7, animals were euthanized, tumors explanted and fixed in formalin as well as preserved by cryoconservation for immunohistochemical stainings (see Fig 1).

### CEUS imaging

CEUS imaging was performed on a clinical ultrasound system (Acuson Sequoia 512, Siemens, Erlangen, Germany), using a conventional 15L8 linear transducer, fixed on a mechanical support to maintain the same imaging position in plane over time and maximize reproducibility (transmit frequency 7 MHz, dynamic range 80 dB, depth 25 mm and time-gain compensation, acoustic focus placed at the largest tumor cross section) [9]. Tumors were imaged in their long axis at baseline (day 0) and follow-up (day 7). Anesthetized rats were fixed in side position and ultrasound coupling gel was applied onto shaved skin. First a B-mode scan of each tumor was performed to visualize subcutaneous tumor expansion. Afterwards cadence pulse sequencing mode (CPS) was set at low acoustic power (mechanical index (MI) = 0.16) to limit the amount of MB destruction. Video clips acquisition was initiated immediately before injection of BR55, continued during the first minute and afterwards minute-by-minute up to 10 minutes after contrast injection (frame rate 16 Hz), measuring wash-in and wash-out of the MB in the tumor. VEGFR2-specific binding was assessed at 8 and 10 minutes after BR55 injection, when circulating MB disappeared from the general circulation but the tumor tissue was still highlighted, indicating specific accumulation of targeted MB on VEGFR2-expressing endothelium.

### Data postprocessing

**Fig 2:**
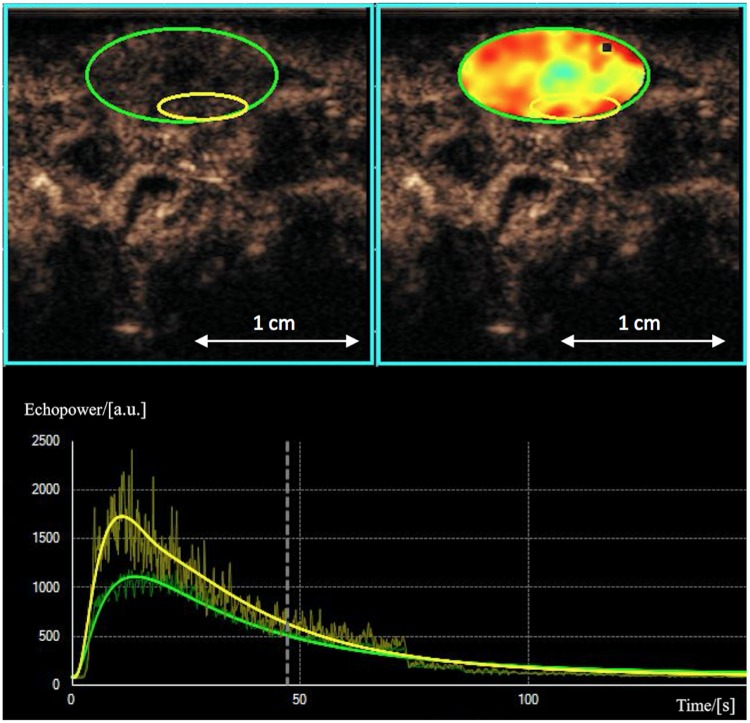
ROI selection. Representative CEUS images of a rat after 7 days of regorafenib therapy with a subcutaneous tumor xenograft (green) and blood volume parameter map with a ROI in a hypervascular vital tumor site of the outer rim (yellow). Corresponding signal-intensity-versus-time-curves are shown below.

CEUS data were post-processed using dedicated software on an external workstation with Vuebox^®^ (Bracco Suisse SA, Geneva, Switzerland) by drawing a region of interest (ROI) into a highly perfused region of the vital outer rim of the tumor. This software is designed to quantify contrast enhancement within the ROI, expressed as relative echo-power values proportional to MB concentration and assessed in arbitrary units (a.u.) [11] [9,13] (see Fig 2). Target parameters were wash-in area under the curve (WiAUC) in the early vascular phase, to provide information about blood flow and volume [16,25], as well as measured signal intensity after 8 minutes (SI_8min_) and 10 minutes (SI_10min_) p.i., as surrogate parameters of VEGFR2-specific binding of MB in the late molecular phase.

### Microbubbles

BR55 (Bracco Suisse SA, Geneva, Switzerland) is an experimental intravenous ultrasound contrast agent comprising VEGFR2-specific targeted MB. The VEGFR2-binding lipopeptide was prepared by conjugation of a heterodimer peptide to the amino group of DSPE-PEG2000-NH2 and incorporated into the phospholipid-based MB formulation. The resulting product (BR55) is a lyophilisate in a septum-sealed vial. The gas phase in the vial is a mixture of perfluorobutane and nitrogen [9,12,26]. Before use BR55 has to be reconstituted by injection of 2 mL of a 5% glucose solution through the septum of the vial. After dissolution, the resulting MB suspension is ready for use. Mean diameter of BR55 MB is 1.5 μm with a mean concentration of 2 x 10^9^ MB per mL. The number of lipopeptide molecules per MB is approximately 4 x 10^5^ [9,19]. Circulation time of BR55 is nearly 4 minutes [20]. BR55 was administrated to each rat at a dose of 50 μL followed by a 150 μL saline bolus by standardized manual intravenous bolus injection through a tail vein catheter.

### Multiparametric MRI protocol

MRI was performed on a clinical 3 Tesla MRI scanner (Magnetom Skyra, Siemens Healthcare, Erlangen, Germany) with the anesthetized rats in supine position using a four-channel small flex coil (Siemens Healthcare, Erlangen, Germany). For the assessment of tumor morphology, T2-weighted MR images were acquired using a 2D Turbo Spin Echo sequence (TR/TE: 5470/91ms) with 0.3 x 0.3 mm in-plane resolution resulting in a matrix size of 192 x 192 and slice thickness of 1.5 mm. Subsequently, a view-sharing sequence (TWIST, fast view-sharing gradient-recalled echo time-resolved angiography with stochastic trajectories, Siemens Healthcare, Erlangen, Germany) with high temporal and spatial resolution [27] was started for acquisition of the pre-contrast baseline and subsequent contrast medium bolus tracking with acquisition of n = 300 datasets resulting in an time to acquisition of 10:13 min (Sequence details: TR/TE: 6.34/2.11 ms; flip angle 40°; matrix size 128 x 128; field of view 50 x 50 mm^2^; spatial resolution, 0.39 x 0.39 x 3.0 mm^3^). For standardized contrast media administration, an automated bolus of 0.1 mmol/kg body-weight of gadobutrol (Gadovist^®^ 0.1 mmol/ml injection solution, Bayer, Leverkusen, Germany) followed by a saline bolus of 0.5 mL was applied via tail vein catheter using a dedicated small animal contrast media injection system (PHD2000 Series, Harvard Apparatus, Holliston, MA).

### MRI data processing and kinetic analysis

Data sets were analyzed on an external workstation using a dedicated post-processing software (PMI, Platform for Research in Medical Imaging version 0.4), written in-house in IDL 8.3 (ITT Visual Information Solutions, Boulder, CO). Corresponding to CEUS measurements, a tissue region of interest was placed over the tumor periphery using semi-quantitative area under the curve (AUC) maps [28]. Tumor periphery was defined as the outer rim of the tumor (3 mm) as a representative region of viable tumor tissue which is less affected by elevated interstitial pressure and necrosis compared to the tumor center [29]. An additional blood ROI was drawn into the lumen of the intrahepatic part of the inferior vena cava in order to gain an arterial input function (AIF). Signal-intensity-versus-time-curves were extracted for the tumor tissue ROI and the AIF. Tracer concentrations were approximated in accordance to relative enhancement S/S_0-1_ (S is signal intensity; S_0_ is signal intensity prior to arrival of the contrast agent). A two-compartment exchange model [30,31] was fitted to the MRI data and two independent quantitative parameters were selected: tumor plasma flow PF (mL/100mL/min), as a parameter of tumor perfusion, and tumor plasma volume PV (mL/100mL = %), as a parameter of tumor vascularity.

### Immunohistochemistry

The formaldehyde-fixed and paraffin-embedded tumor tissue was stained with regard to the following aspects of tumor pathophysiology: tumor necrosis (H&E), microvascular density (CD31), tumor cell proliferation (Ki-67), tumor apoptosis (TUNEL) and VEGFR2 expression. Tissue sections were dewaxed and rehydrated following standard procedures including pre-heating at 60°C and washing in xylene substitute (Neo-Clear^®^, Merck Millipore, Darmstadt, Germany) with rehydration in a graded series of ethanol (100%, 96%, 90% and 70% ethanol) followed by double distilled water. Afterwards dedicated staining procedures were initiated.

#### Hematoxylin and eosin (H&E)

For the assessment of necrotic tumor areas H&E stainings were performed. Paraffin was removed and tumor tissue sections were incubated in Mayer´s hemalum solution (Merck Millipore, Darmstadt, Germany) for 5 minutes and purged by distilled water. Subsequently tissue sections were incubated for 10 minutes under running water and purged again briefly by distilled water, stained in eosin solution for a few seconds and dehydrated in a graded series of ethanol (70%, 90%, 96% and 100% ethanol). Afterwards the tissue sections were incubated for 5 minutes in NeoClear^®^ (Merck Millipore, Darmstadt, Germany) twice. Finally the sections were embedded with EuKitt^®^ (Sigma-Aldrich, St. Louis, MO). Results were quantified as the percentage of necrotic tissue fraction in 10 random fields at 200x magnification.

#### CD31

For immunohistochemical assessment of tumor microvascular density, tumor sections were incubated with a polyclonal rabbit anti-CD31 primary antibody (Abcam ab28364 1:50, Cambridge, United Kingdom) overnight. Further work-up of tissue samples was performed using the EnVision+ System HRP (AEC) (DAKO Diagnostika, Hamburg, Germany) according to the manufacturer´s instruction. Counterstaining was performed using Mayer´s hemalum (Merck Millipore, Darmstadt, Germany) and slides were covered with Kaiser´s Glycerin Gelatine (Merck Millipore, Darmstadt, Germany). Tumor microvessels were quantified as the average number of endothelial cells in 10 random fields at 200x magnification [32].

#### Ki-67

*Ki-67*-specific monoclonal rabbit anti-human antibody (SP6, Abcam ab16667 1:100, Cambridge, United Kingdom) was used to quantify tumor cell proliferation. The tissue was demasked in 1 x citrate buffer (pH = 6.0) using microwave irradiation at 600 W. After washing the slides in ddH_2_O and TBS-Tween (0.05%) the multi-step kit EnVision+ System HRP (DAB) (DAKO Diagnostika, Hamburg, Germany) was used for the staining following the manufacturer’s instructions. Counterstaining was performed using Mayer´s hemalum (Merck Millipore, Darmstadt, Germany) and slides were covered with Kaiser´s Glycerin Gelatine (Merck Millipore, Darmstadt, Germany). Results were quantified as the average number of proliferating cells in 10 random fields at 200x magnification.

#### TUNEL

For the quantification of apoptotic cells *Terminal deoxynucleotidyl transferase–mediated nick end labeling* (TUNEL) staining was performed with a commercially available apoptosis detection kit (In Situ Cell Death Detection Kit, Roche Diagnostics AG, Risch, Switzerland) according to the manufacturer’s instructions. Tissue samples were subsequently analyzed using fluorescence microscopy with a standard fluorescent filter set at 520 ± 20 nm. Results were expressed as the average number of apoptotic cells in 10 random fields at 200× magnification (cells per high-power field).

#### VEGFR2

The expression of VEGFR2 was analyzed using a VEGFR2-specific monoclonal rabbit anti-human antibody (1:50; Cell Signalling, Cambridge, UK). Following standard procedures, tissue samples were dewaxed, rehydrated and subsequently demasked in target-retrieval solution (DAKO, Hamburg, Germany) using microwave irradiation at 600 W. After overnight incubation with the primary antibody, tissue samples were further processed using the EnVision+ System HRP (DAB) (DAKO Diagnostika, Hamburg, Germany). VEGFR2-positive stained tumor vessels were quantified as the number in 10 random high-power fields at a magnification of 200x.

### Statistical analysis

Continuous variables are presented as means with standard deviations (mean ± SD). For intergroup comparisons of CEUS parameters, MRI perfusion parameters and immunohistochemical values between the treatment and the control group, the Mann-Whitney-U-test was used. For intragroup comparisons of CEUS and MRI perfusion parameters between baseline (day 0) and follow-up (day 7) a Wilcoxon-signed-rank test was applied. Correlations between CEUS and MRI parameters as well as between CEUS, MRI and immunohistochemistry were evaluated by Spearman’s rank correlation coefficients (Spearmans rho = ρ). P-values < 0.05 were considered statistically significant. All analyses were performed with a dedicated statistics software (SPSS Version 23 for Microsoft Windows, IBM, Armonk, NY).

## Results

The experimental protocol, including multimodality imaging with CEUS and DCE-MRI, was successfully completed in n = 17 animals. In n = 3 animals the ultrasound and in n = 1 animal the MRI measurements could not be accomplished due to technical issues. N = 1 animal of the control group had to be excluded due to anesthesia complications. Animals were assigned randomly to either the therapy or the control group with no significant differences (p > 0.05) in tumor size as well as in CEUS and MRI values at baseline.

### Tumor size

Tumor size was assessed by volumetric MRI measurements at baseline and follow-up. There were no significant differences (p > 0.05) in mean tumor sizes between the therapy and the control group on day 0 (524 ± 184 mm^3^ vs. 571 ± 289 mm^3^) and on day 7 (531 ± 352 mm^3^ vs. 651 ± 311 mm^3^), as well as no significant changes (p > 0.05) in tumor growth between day 0 and day 7 in the therapy (from 524 ± 184 mm^3^ to 531 ± 352 mm^3^) or in the control group (from 571 ± 289 mm^3^ to 651 ± 311 mm^3^) (see S1 Table).

### CEUS

**Fig 3:**
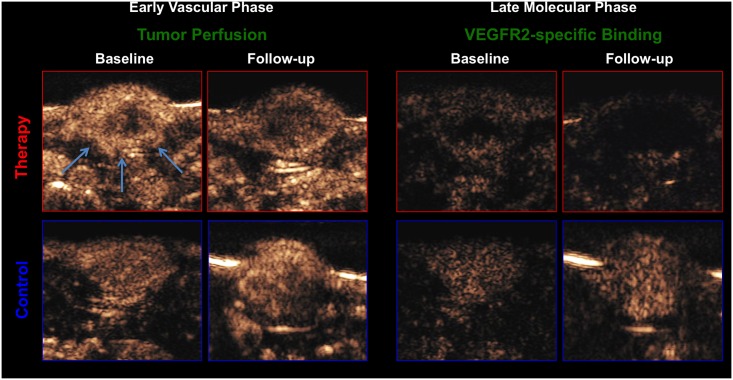
Representative CEUS images of a rat with a subcutaneous tumor xenograft (white arrows) under treatment. Therapy group (red) and control group (blue) at baseline and follow-up. Left side: early vascular phase with BR55 as a functional imaging biomarker; right side: late phase of VEGFR2-specific binding with BR55 as a molecular imaging biomarker 8 minutes after contrast injection. Note the significant lower number of circulating microbubbles in the early vascular phase as well as the significant lower number of bound microbubbles in the late phase at follow-up in the therapy group, compared to baseline and compared to the control group.

In CEUS with BR55, functional parameters of tumor microcirculation were assessed during an early vascular phase and VEGFR2-specific binding of the MB during a late molecular phase up to 10 min p.i. (see Fig 3).

**Fig 4:**
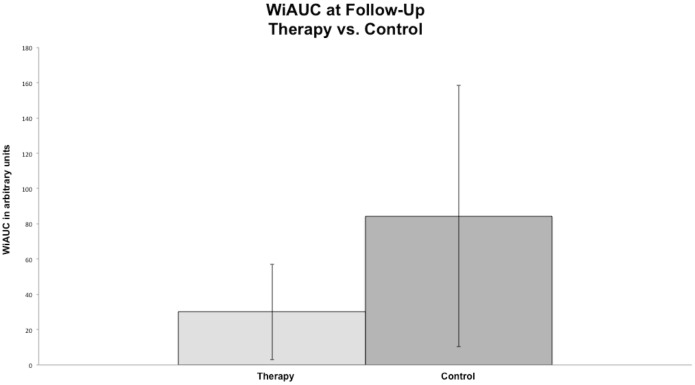
Column charts of WiAUC in the therapy and control group at follow-up. Note the significant difference (p < 0.05) between the mean values of WIAUC between the therapy and the control group at the follow-up.

In the therapy group a significant decrease of tumor microcirculation was observed following the one-week treatment course with regorafenib, assessed by a significant decline of WiAUC from 116,989 ± 77,048 a.u. on day 0 to 30,076 ± 27,095 a.u. on day 7 (p = 0.005). In the control group no significant changes (p = 0.093) in WiAUC (from 133,932 ± 65,960 a.u. on day 0 to 84,316 ± 74,144 a.u. on day 7) were noted. Moreover there were significantly lower values of WiAUC (p = 0.009) in the therapy than in control group on day 7 (see Fig 4 and Table 1).

**Table 1:** Individual functional CEUS values.

Animal No.	WiAUC [a.u.]	
	Day 0	Day 7
THERAPY GROUP		
1	n/a	n/a
2	102,709	29,476
3	50,150	39,784
4	47,518	7,779
5	282,576	5,463
6	200,592	19,290
7	69,803	31,530
8	82,944	7,070
9	66,663	39,336
10	94,354	22,951
11	172,584	98,085
Mean	116,989*	30,076*^/^^#^
SD	77,048	27,095
CONTROL GROUP		
12	n/a	n/a
13	92,930	69,166
14	232,554	85,876
15	117,899	261,323
16	n/a	n/a
17	116,438	78,529
18	53,246	23,437
19	118,856	48,542
20	102,021	49,414
21	237,511	58,243
Mean	133,932	84,316^#^
SD	65,960	74,144

Individual functional CEUS values of tumor microcirculation in subcutaneous human colon carcinoma xenografts at baseline and follow-up in the therapy and in the control group. Note the significant decrease of WiAUC between day 0 and day 7 in the therapy group as well as the significantly lower mean WiAUC values in the therapy than in the control group at follow-up.

* significant difference (p < 0.01) between baseline and follow-up

^#^ significant difference (p < 0.01) between therapy and control group

n/a = not available due to technical issues

**Fig 5:**
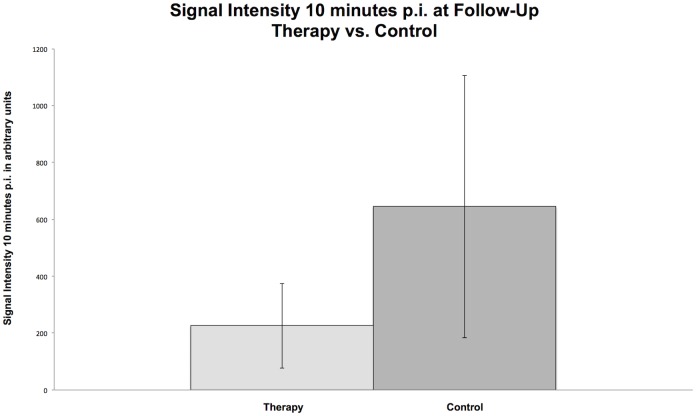
Column charts of SI_10min_ in the therapy and control group at follow-up. Note the significant difference (p < 0.05) between the mean values of SI_10min_ between the therapy and the control group at the follow-up.

In the late molecular phase, a significant decline in tumor signal intensity between baseline and follow-up was observed in the therapy group (SI_8min_: from 2,781 ± 1,816 to 283 ± 191 a.u., p = 0.005; SI_10min_: from 2,412 ± 1,657 to 226 ± 149 a.u., p = 0.005) and in the control group (SI_8min_: from 3,659 ± 2,588 to 802 ± 460 a.u., p = 0.012; SI_10min_: from 3,164 ± 2,283 to 645 ± 461 a.u., p = 0.012). However, significantly lower SI_8min_ (283 ± 191 vs. 802 ± 460 a.u., p = 0.0062) and SI_10min_ (226 ± 149 vs. 645 ± 461 a.u., p = 0.009) values were detected in the therapy than in the control group on day 7 (see Fig 5 and Table 2).

**Table 2:** Individual molecular CEUS values.

Animal No.	SI_8min_ [a.u.]		SI_10min_ [a.u.]	
	Day 0	Day 7	Day 0	Day 7
THERAPY GROUP				
1	n/a	n/a	n/a	n/a
2	2,048	533	1,794	502
3	2,012	173	1,813	118
4	1,479	175	1,257	143
5	6,471	99	5,348	84
6	5,849	188	5,642	138
7	2,640	226	2,277	181
8	1,835	166	1,549	145
9	1,867	467	1,578	365
10	2,089	157	1,749	140
11	1,515	649	1,110	433
Mean	2,781*	283*^/^^#^	2,412*	226*/#
SD	1,816	191	1,657	149
CONTROL GROUP				
12	n/a	n/a	n/a	n/a
13	4,055	996	3,562	835
14	4,695	1,667	4,695	1,528
15	9,235	592	7,858	446
16	n/a	n/a	n/a	n/a
17	1,815	1,224	1,544	1,010
18	1,246	695	1,001	607
19	2,325	460	1,920	348
20	1,817	328	1,413	223
21	4,086	453	3,316	166
Mean	3,659**	802**	3,164**	645**/#
SD	2,588	460	2,283	461

Individual CEUS signal intensity values of VEGFR2-specific binding in the late phase at baseline and follow-up in the therapy and in the control group. Note the significant decrease of signal intensity at 8 minutes (SI_8min_) and 10 minutes (SI_10min_) post injection between day 0 and day 7 in the therapy and the control group. However, significantly (p < 0.01) lower mean SI_8min_ and SI_10min_ was observed in regorafenib-treated animals compared to the control group.

* significant difference (p < 0.01) between baseline and follow-up

** significant difference (p < 0.03) between baseline and follow-up

^#^ significant difference (p < 0.01) between therapy and control group

n/a = not available due to technical issues

### DCE-MRI

The two-compartment model fit the data well in all experiments. In the regorafenib-treated therapy group mean PF declined significantly (p = 0.003) from 147 ± 58 mL/100 mL/min at baseline to 71 ± 15 mL/100 mL/min at follow-up. In the control group no significant (p = 0.515) changes were observed between day 0 and day 7 (from 108 ± 24 to 113 ± 31 mL/100 mL/min). Individual values showed a unidirectional decline of tumor perfusion in the therapy group and a heterogeneous development in the control group. Mean PV decreased significantly (p = 0.040) over the one-week treatment course from 13 ± 3% at baseline to 9 ± 4% at follow-up. In the control group no significant change (p = 0.514) of mean plasma volume was observed between baseline and follow-up (from 14 ± 6 to 16 ± 8%). Additionally significantly lower values of PF (71 ± 15 vs. 113 ± 31 mL/100 mL/min, p = 0.002) and PV (9 ± 4 vs. 16 ± 8%, p = 0.012) were measured in treated than in non-treated animals on day 7 (see Table 3).

**Table 3:** Individual DCE-MRI values.

Animal No.	PF [mL/100mL/min]		PV [%]	
	Day 0	Day 7	Day 0	Day 7
THERAPY GROUP				
1	140	48	9	6
2	96	79	9	3
3	114	86	10	11
4	195	63	13	10
5	213	77	10	17
6	249	86	15	11
7	130	78	12	9
8	123	64	12	8
9	81	47	13	7
9	201	90	14	5
11	78	61	20	7
Mean	147*	71*^/^^#^	13*	9*^/^^#^
SD	58	15	3	4
CONTROL GROUP				
12	155	99	8	10
13	93	114	8	15
14	94	112	8	7
15	110	139	17	26
16	140	124	14	10
17	n/a	n/a	n/a	n/a
18	107	46	24	14
19	86	156	20	12
20	102	125	15	28
21	86	100	12	21
Mean	108	113^#^	14	16^#^
SD	24	31	6	8

Individual DCE-MRI parameters of tumor microcirculation with tumor plasma flow (PF; mL/100mL/min) and plasma volume (PV; %) at baseline and follow-up in the therapy and in the control group. Note the significant decrease of tumor microcirculatory parameters PF and PV between day 0 and day 7 as well as the significantly lower mean values of PF and PV in the therapy than in the control group at the follow-up.

* significant difference (p < 0.05) between baseline and follow-up

^#^ significant difference (p < 0.03) between therapy and control group

n/a = not available due to technical issues

### Immunohistochemistry

**Fig 6:**
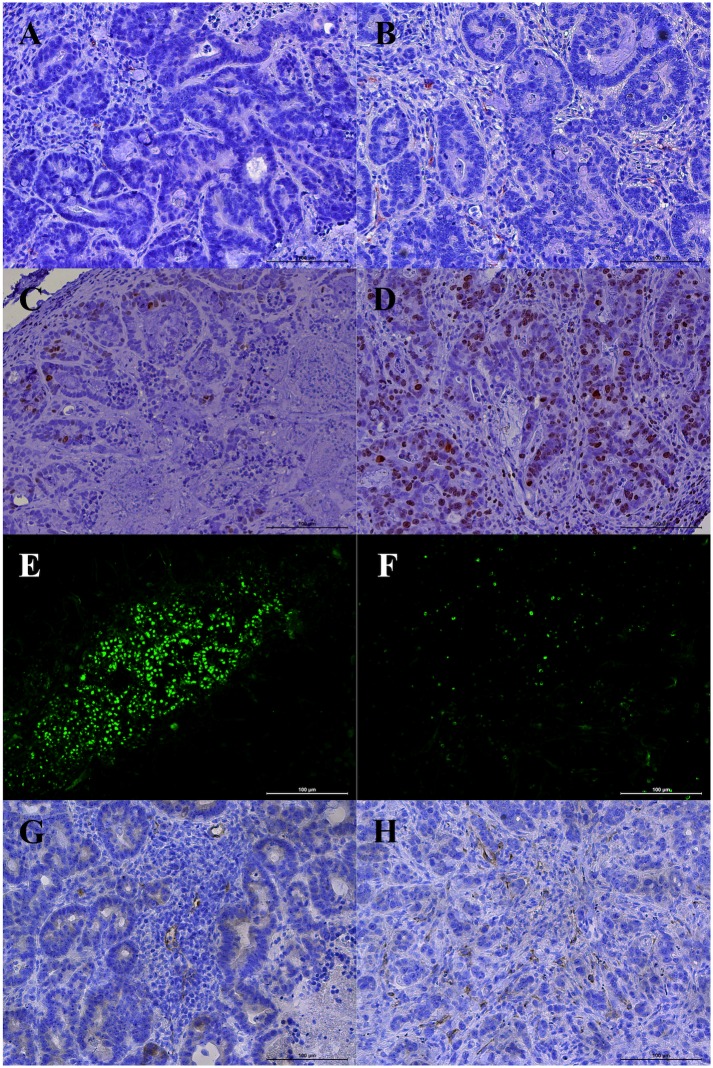
Representative immunohistochemical stainings. CD31 (A, B; microvascular density), Ki-67 (C, D; cell proliferation), TUNEL (E, F; apoptosis) and VEGFR2 expression (G,H) in the therapy (left column: A, C, E, G) and in the control group (right column: B, D, F, H). Note the significant lower (p < 0.01) microvascular density, tumor cell proliferation and VEGFR2 expression and the significant higher (p < 0.01) apoptosis rate in the therapy group. Scale bar in images represents 100 μm.

Significantly (p < 0.05) more necrotic tumor tissue was observed in regorafenib treated animals than in the placebo group (58.6 ± 14.2% vs. 18.3 ± 6.4%). Significant anti-angiogenic effects of regorafenib were observed in the investigated colon carcinoma xenografts with a significantly lower (p < 0.001) microvascular density in regorafenib-treated than in non-treated animals (8.1 ± 3.0 vs. 20.8 ± 5.7), quantified by CD31 stainings. Significant anti-proliferative effects of regorafenib were noted in Ki-67 stainings with a significantly lower (p = 0.004) number of proliferating cells in the therapy than in the control group (318.7 ± 94.0 vs. 468.0 ± 133.8). Pro-apoptotic effects of regorafenib were quantified by TUNEL stainings with a significantly higher (p = 0.003) number of apoptotic cells in the therapy than in the control group (627.7 ± 194.0 vs. 357.6 ± 192.0). VEGFR2 stainings showed a significantly lower (p < 0.001) VEGFR2 expression in regorafenib-treated than in placebo-treated animals (7.2 ± 1.8 vs. 17.8 ± 4.6) (see Fig 6 and Table 4).

**Table 4:** Individual immunohistochemical values.

Animal No.	HE	CD31	Ki-67	TUNEL	VEGFR2
THERAPY GROUP					
1	66.9	7.7	286.6	345.9	6.3
2	61.9	10.2	232.3	858.3	9.5
3	62.7	10.2	349.7	613.5	8.2
4	33.7	10.1	412.1	436.4	8.2
5	70.7	4.0	265.3	739.5	5.9
6	55.0	9.7	233.8	781.4	7.5
7	57.7	9.8	330.8	936.4	5.8
8	35.2	3.7	216.8	582.1	3.3
9	50.8	3.0	289.2	574.8	7.7
10	73.1	10.4	537.3	n/a	9.1
11	76.4	9.8	352.2	858.2	7.0
Mean	58.6*	8.1*	318.7*	672.7*	7.2*
SD	14.2	3.0	94.0	194.0	1.8
CONTROL GROUP					
12	21.9	30.2	338.7	597.2	16.9
13	16.8	23.5	449.6	464.6	24.6
14	18.3	19.5	300.8	390.5	16.8
15	14.3	18.8	597.9	152.8	16.0
16	22.5	27.4	370.9	480.4	20.1
17	11.8	10.5	583.5	326.8	18.8
18	8.3	22.5	414.0	34.6	22.3
19	31.6	14.3	592.5	423.6	15.3
20	18.3	21.0	352.6	570.8	7.6
21	19.0	20.3	679.5	134.9	19.4
Mean	18.3*	20.8*	468.0*	357.6*	17.8*
SD	6.4	5.7	133.8	192.0	4.6

Individual values for the investigated histological and immunohistochemical parameters of tumor necrosis (HE), tumor microvascular density (CD31), tumor cell proliferation (Ki-67), tumor cell apoptosis (TUNEL) and VEGFR2 expression in the therapy and in the control group. Note the significantly higher necrosis rate (%) and higher number of apoptotic cells as well as the significantly lower tumor vascularity, tumor cell proliferation and VEGFR2 expression in the regorafenib-treated therapy group.

* significant difference (p < 0.01) between therapy and control group

n/a = not available due to technical issues

### Correlations between CEUS and immunohistochemistry

CEUS perfusion parameter WiAUC showed moderate, but significant correlations to CD31 (ρ = 0.053; p = 0.024), to Ki-67 (ρ = 0.53; p = 0.025), as well as to VEGFR2 stainings (ρ = 0.48; p = 0.042) (see Table 5).

**Table 5:** Correlations between functional CEUS values and immunohistochemistry.

Correlation	Spearman ρ	p
**WiAUC/CD31**	0.53*	0.024
**WiAUC/Ki-67**	0.53*	0.025
**WiAUC/VEGFR2**	0.48*	0.042

*Significant correlations between functional CEUS parameter of tumor microcirculation WiAUC and immunohistochemical markers of tumor microvascular density (CD31), tumor cell proliferation (Ki-67) and VEGFR2 expression.

**Fig 7:**
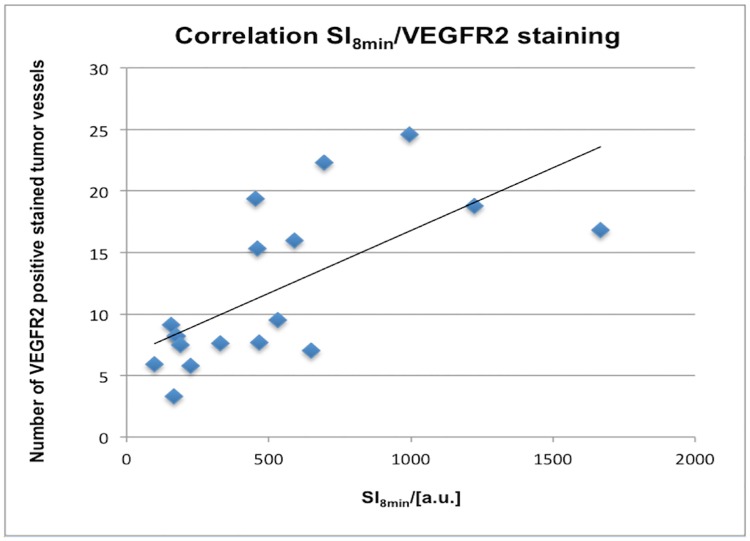
Line regression analysis for the correlation between CEUS parameter of VEGFR2-specific binding and immunohistochemical VEGFR2 expression. Note the significant correlation between the values of SI_8min_ and the number of VEGFR2 positive stained vessels (Spearman´s ρ = 0.66, p = 0.003) for the therapy and the control group.

In the late VEGFR2-specific phase, significant moderate to good correlations between molecular CEUS parameters and immunohistochemistry were observed: immunohistochemical VEGFR2 expression correlated significantly with SI_8min_ (ρ = 0.66; p = 0.003) and with SI_10min_ (ρ = 0.62; p = 0.006) (see Fig 7). Additionally microvascular density (CD31) showed significant moderate correlations to SI_8min_ (ρ = 0.54; p = 0.022) and to SI_10min_ (ρ = 0.53; p = 0.025) (see Table 6).

**Table 6:** Correlations between molecular CEUS values and immunohistochemistry.

Correlation	Spearman ρ	p
**SI**_**8min**_**/CD31**	0.54*	0.022
**SI**_**10min**_**/CD31**	0.53*	0.025
**SI**_**8min**_**/VEGFR2**	0.66*	0.003
**SI**_**10min**_**/VEGFR2**	0.62*	0.006

*Significant, moderate correlations between molecular CEUS parameters of VEGFR2-specific binding (SI_8min_ and SI_10min_) and immunohistochemical CD31 and VEGFR2 expression.

### Correlations between DCE-MRI and immunohistochemistry

Moderate, significant correlations were observed between MRI perfusion parameters and immunohistochemistry: PF correlated significantly with CD31 (ρ = 0.62; p = 0.003), Ki-67 (ρ = 0.49; p = 0.030) and with VEGFR2 (ρ = 0.46; p = 0.042). PV correlated significantly with Ki-67 (ρ = 0.49; p = 0.030).

### Correlations between CEUS and DCE-MRI parameters

A significant, moderate correlation was observed between CEUS parameter WiAUC and MRI parameter PF (ρ = 0.51; p = 0.035).

## Discussion

The present study investigated the potential of CEUS with BR55 for the non-invasive monitoring of regorafenib therapy effects on experimental colorectal carcinoma xenografts in rats and its applicability to generate functional and molecular imaging biomarkers of therapy response correlated to DCE-MRI and validated with multiparametric immunohistochemistry. Our results provide evidence that multiparametric CEUS with BR55 can be applied for the detection of early regorafenib therapy effects based on different functional and molecular parameters of tumor pathophysiology.

### CEUS: assessment of tumor microcirculation and VEGFR2-specific imaging

Significant changes of tumor microcirculation were observed with a significant decline of WiAUC between day 0 and day 7 under therapy. We noted no significant changes in WiAUC in the control group, under the constraint that a mild outlier favors this result. If we exclude this mild outlier, the control group would also have a significant decrease of WiAUC. Nevertheless we observedsignificantly lower values of WiAUC in the therapy group than in the control group at the follow-up, signalling the regorafenib therapy effect. This is in accordance with Zocco et al. who applied CEUS with untargeted microbubbles to investigate patients with advanced hepatocellular carcinoma under sorafenib therapy in a clinical study, assessing parameters of microcirculation including AUC and observed a significant decrease of AUC following 15 days of sorafenib therapy, associated with a longer survival and with a significant correlation to progression free survival [33]. Differing from our study, Zocco et al. applied a non-targeted intravascular ultrasound contrast agent, which, however, resembles BR55 in bubble size and kinetics providing similar measures of tumor microcirculation and a similar wash-in-phase of the MB uptake as non-targeted agents with intravascular kinetics and distribution to the same tumor regions [13,34,35].

With regard to the assessment of VEGFR2 expression in the molecular late phase, on day 7 significantly lower SI_8min_ and SI_10min_ values were observed in the therapy group compared to the control group at follow-up, reflecting a significantly lower number of MB bound to VEGFR2 on the tumor endothelial surface in tumors following regorafenib therapy. This is in accordance with Baetke et al., who showed in their study with heterotopic squamous cell carcinoma xenografts in nude mice under anti-VEGF antibody therapy 8 minutes after BR55 MB injection a significant lower amount of specifically bound BR55 MB in treated tumors compared to the control group on day 4, day 7 and day 14 [21]. Corresponding to our results, Pysz et al. reported a significant difference of targeted imaging signal using CEUS and BR55 already 24h after initiation of anti-VEGF-antibody therapy in human colon cancer xenografts in mice, which remained significantly lower in treated than in untreated animals during 5 days of observation [10]. Differing from our findings, Pysz and colleagues only observed a significant decrease of targeted imaging signal in treated and not in untreated animals, however using a shorter treatment interval of only 24h.

Our observation, that SI_8min_ and SI_10min_ decreased significantly between day 0 and day 7 not only in the regorafenib-treated therapy but also in the untreated control group is paralleled by findings of Baron Toaldo et al., who reported a progressive reduction of the differential targeted enhancement (dTE = SI before destruction of MBs–SI after destruction of MBs) using BR55-enhanced CEUS in sorafenib-treated as well as in untreated animals [11]. As possible explanation for this unexpected finding Baron Toaldo and colleagues discussed the occurrence of large necrotic areas in untreated control tumors as a result of rapid uninhibited tumor growth lacking a correspondingly large enough vasculature with consecutively lower BR55 binding in tumors of the control group. The authors argued that the production of VEGF is dependent on tumor cell mass and that the reduction of effective vital tumor mass in rapidly growing tumors might be responsible for the transient reduction in vascular proliferation and VEGFR2 expression with decreased BR55 binding [11]. Consequently, the development of necrosis may be considered a confounding cause of the reduction of dTE also in treated tumors. To investigate this potential confounding factor in our study, H&E stainings were performed for the assessment of necrotic tumor tissue in treated and untreated tumors and revealed significantly larger necrotic areas in the regorafenib-treated compared to the control group. However, significant central tumor necrosis was also observed in the untreated animals of the control group, as a potential confounding factor for the significant decrease of VEGFR2-specific MB binding in both groups. Therefore, in contrast to the study of Baron Toaldo et al., who quantified ROIs enclosing the whole tumor, in the present study ROIs were selected over the vital outer rim of therapy and control tumors to avoid tumor necrosis and areas of elevated interstitial pressure with consecutively altered contrast media kinetics with the aim to exclude tumor necrosis as potential confounding factor for the observed reduction of VEGFR-2 expression. The observed moderate, yet significant reduction of VEGFR-2 MB binding in tumors of the control group between day 0 and day 7 remains therefore unclear and warrants further investigation of the specific kinetics of VEGFR-2 targeting MB in untreated tumors. Nonetheless, the significantly lower number of MB bound to VEGFR2 on the tumor endothelial surface in tumors following regorafenib therapy on day 7 allowed for the reliable identification of responding, regorafenib-treated tumors compared to the control group.

### DCE-MRI validation

During a one-week treatment protocol with regorafenib, a significant decrease of tumor perfusion (PF) and tumor vascularity (PV) was observed between day 0 and day 7 in the therapy group, with no significant changes detected in the control group. These results are in accordance with several preclinical and clinical studies in the literature [28,36,37]. Intraindividual comparisons of early vascular CEUS and DCE-MRI perfusion parameters revealed only moderate, but significant correlations for WiAUC (CEUS) and PF (DCE-MRI). Correlations were possibly hampered by the circumstance that similar, but not identical parameters of tumor microcirculation were acquired by CEUS and DCE-MRI, reflecting preferably similar but not identical pathophysiological processes, as well as differences in scanning and slice positions. Although the time between both examinations was kept to a minimum in our study, we have had leveled experimental conditions with no synchronous acquisition of CEUS and DCE-MRI parameters, that would possibly broaden concordance between CEUS and MRI parameters.

### Immunohistochemical validation

Immunohistochemical analysis revealed significant anti-angiogenic, anti-proliferative and pro-apoptotic effects of regorafenib on colorectal carcinoma xenografts, as well as a significantly reduced expression of VEGFR2. CEUS parameter of tumor microcirculation WiAUC in the early vascular phase showed moderate, significant correlations to immunohistochemical CD-31, Ki-67 and VEGFR2 stainings. This is in accordance with Forsberg et al., who demonstrated a significant correlation of histological tumor vascularity and CEUS measurements with non-targeted ultrasound contrast media in a recent study with breast tumors, by correlating the ultrasonic fractional breast tumor vascularity (FV = number of color pixels relative to the total number of pixels within the breast mass) and CD31 [38]. SI_8min_ and SI_10min_ in the late molecular phase, as parameters of VEGFR2-specific binding, also showed significant, moderate correlations to immunohistochemical measures of VEGFR2 expression and to microvascular density (CD31). This is in accordance with Bzyl et al. who discussed in their study with two differently aggressive breast carcinoma xenografts in mice, that the relative amount of receptor-bound MBs corresponds to immunohistochemical differences in tumor microvascular density (CD31) as well as to VEGFR2 expression concluding that BR55 reflects the VEGFR2 status in tumors and can be applied as non-invasive surrogate parameter of tumor angiogenesis [12]. Similarly, Baron Toaldo et al. concluded in his study in mice with HCC xenografts under sorafenib therapy, that the investigated CEUS parameter of VEGFR2 specific binding (dTE) on day 14 is significantly related to immunohistochemically expressed VEGFR2 levels [11].

In consideration of the reported findings, the significant correlations to the immunohistochemical standard of reference in our study support our hypothesis that the investigated parameters of tumor microcirculation indeed reflect processes of tumor pathophysiology and may therefore be applicable as non-invasive imaging biomarkers of therapy response to regorafenib. It furthermore indicates the potential of CEUS to provide non-invasive imaging biomarkers of tumor angiogenesis [14,39–41].

### Limitations

Our study results are limited in several aspects. Firstly, our study investigated only one tumor-therapy-combination in a heterotopic xenograft model of colon cancer with only limited translational relevance to orthotopic tumor pathophysiology in humans [42]. Nevertheless other experiments demonstrated the clinical potential of CEUS for monitoring changes in tumor vascularisation secondary to anti-angiogenetic cancer treatments [16]. Secondly we observed only a short period of time without determining clinical endpoints such as overall survival of the animals, but our preclinical study was focused on novel imaging biomarkers of an early therapy response, that allow a reliable and timely differentiation of responders from non-responders. To validate our imaging results, several established immunohistochemical parameters were investigated, reflecting central and representative aspects of tumor pathophysiology under anti-angiogenic therapy, as surrogate markers of therapy response [1] Morevover, as mentioned before, Zocco et al. observed in patients with advanced hepatocellular carcinoma under sorafenib therapy an association of a significant decrease of ultrasound perfusion parameters under therapy with a longer survival and a significant correlation to progression free survival [33]. Thirdly analysis was limited to a single, central plane of the tumor. In CEUS as well as in DCE-MRI it is not completely avoidable that animals are scanned in slightly different planes at baseline and follow-up due to changes in tumor size during the treatment course with possible influence on parameters of tumor microcirculation and VEGFR2 binding. To minimize these differences and to maximize reproducibility we used a mechanical support for fixing the animals and care was taken to reposition the imaging plane on the largest, central cross section of the tumor in order to maintain similar scanning positions and slices during each measurement [11]. In this case the use of three-dimensional CEUS would be helpful, especially in the evaluation of heterogeneous tumors by providing a volumetric assessment of parameters, which will allow sampling not only of a small anatomical area but a more complete assessment of tumor vascularity [14,43,44]. Thirdly it has been demonstrated in former experiments, that BR55 binds to VEGFR2 with high affinity and specificity [9,10,13]. However, no dedicated *in vivo* blocking studies were performed to discriminate between endothelium- and possible extracellular cell-bound BR55, as BR55 has an intravascular distribution profile without relevant extravasation [9,10,12,20].

## Conclusion

In conclusion, our results indicate that multiparametric CEUS with VEGFR2-targeted MB allows for monitoring functional and molecular therapy effects of regorafenib on the investigated experimental colorectal adenocarcinomas in rats. Experiments revealed a significant decline of CEUS and DCE-MRI perfusion parameters in the therapy group between baseline and follow-up as well as a significant reduction of VEGFR2 binding MB under therapy paralleled by a significantly reduced expression of VEGFR2 assessed by immunohistochemistry. CEUS with VEGFR2-targeted MB offers a comprehensive, multi-facetted and non-invasive characterization of tumor angiogenesis. Our results provide encouraging perspectives for the non-invasive monitoring of microvascular and molecular modifications *in vivo* under anti-angiogenic therapy.

## Supporting Information

S1 Table Tumor Volume. Individual tumor volumes of all colon carcinoma xenografts at baseline and follow-up in the therapy and in the control group. Note there were no significant differences (p > 0.05) in mean tumor sizes between the therapy and the control group on day 0 and day 7, as well as no significant changes (p > 0.05) in tumor growth between day 0 and day 7 in the therapy or in the control group.
(DOCX)
