# Supplementary material for: Contrast-Enhanced Ultrasound with VEGFR2-Targeted Microbubbles for Monitoring Regorafenib Therapy Effects in Experimental Colorectal Adenocarcinomas in Rats with DCE-MRI and Immunohistochemical Validation
Source: PLoS One. 2017 Jan 6;12(1):e0169323. doi: 10.1371/journal.pone.0169323 (PMC5217974; doi:10.1371/journal.pone.0169323)
Supplement: S1 Table — Individual tumor volumes of all colon carcinoma xenografts at baseline and follow-up in the therapy and in the control group. Note there were no significant differences (p > 0.05) in mean tumor sizes between the therapy and the control group on day 0 and day 7, as well as no significant changes (p > 0.05) in tumor growth between day 0 and day 7 in the therapy or in the control group. (DOCX) [file pone.0169323.s001.docx]

| Animal No. | **Tumor Volume [mm^3^]** | | |
| --- | --- | --- | --- |
|  | **Day 0** | | **Day 7** |
| **THERAPY GROUP** | | | |
| 1 | | 209 | 182 |
| 2 | | 473 | 648 |
| 3 | | 447 | 373 |
| 4 | | 355 | 321 |
| 5 | | 516 | 280 |
| 6 | | 812 | 436 |
| 7 | | 658 | 1340 |
| 8 | | 795 | 847 |
| 9 | | 635 | 811 |
| 10 | | 436 | 193 |
| 11 | | 427 | 414 |
| Mean | | 524 | 531 |
| SD | | 184 | 352 |
| **CONTROL GROUP** | | | |
| 12 | | 197 | 292 |
| 13 | | 256 | 534 |
| 14 | | 576 | 714 |
| 15 | | 1019 | 1146 |
| 16 | | 824 | 878 |
| 17 | | 468 | 333 |
| 18 | | 182 | 173 |
| 19 | | 680 | 725 |
| 20 | | 822 | 906 |
| 21 | | 685 | 813 |
| Mean | | 571 | 651 |
| SD | | 289 | 311 |
